# Bridging the Gap Between Computational Efficiency and Segmentation Fidelity in Object-Based Image Analysis

**DOI:** 10.3390/ani14243626

**Published:** 2024-12-16

**Authors:** Fernanda Pereira Leite Aguiar, Irenilza de Alencar Nääs, Marcelo Tsuguio Okano

**Affiliations:** Graduate Program in Production Engineering, Universidade Paulista, Rua Dr. Bacelar 1212, São Paulo 04026-002, SP, Brazil; irenilza.naas@docente.unip.br

**Keywords:** automated feature extraction, computational efficiency, image quantization, image segmentation, machine learning optimization, metadata generation, precision livestock farming, object-based preprocessing

## Abstract

A critical issue in image analysis for analyzing animal behavior is accurate object detection and tracking in dynamic and complex environments. This study introduces a novel preprocessing algorithm to bridge the gap between computational efficiency and segmentation fidelity in object-based image analysis for machine learning applications. The algorithm integrates convolutional operations, quantization strategies, and polynomial transformations to optimize image segmentation in complex visual environments, addressing the limitations of traditional pixel-level and unsupervised methods. This innovative approach enhances object delineation and generates structured metadata, facilitating robust feature extraction and consistent object representation across varied conditions. As empirical validation shows, the proposed preprocessing pipeline reduces computational demands while improving segmentation accuracy, particularly in intricate backgrounds. Key features include adaptive object segmentation, efficient metadata creation, and scalability for real-time applications. The methodology’s application in domains such as Precision Livestock Farming and autonomous systems highlights its potential for high-accuracy visual data processing. Future work will explore dynamic parameter optimization and algorithm adaptability across diverse datasets to further refine its capabilities. This study presents a scalable and efficient framework designed to advance machine learning applications in complex image analysis tasks by incorporating methodologies for image quantization and automated segmentation.

## Simple Summary

This study presents a novel algorithm to enhance object-based image segmentation for machine learning applications. The algorithm achieves precise object delineation by integrating convolutional operations, quantization techniques, and polynomial adjustments and generates rich metadata. This methodology improves feature extraction accuracy and ensures consistent object representation across diverse conditions. The empirical results demonstrate substantial advancements in object identification and classification accuracy, particularly in complex scenarios. Compared to traditional methods, the proposed algorithm offers superior computational efficiency. This research provides a scalable and effective preprocessing pipeline that significantly enhances the performance of machine learning models. Future efforts will focus on optimizing dynamic parameters and extending the algorithm’s application to broader datasets.

## 1. Introduction

A significant challenge in image analysis for studying animal behavior is achieving precise object detection and tracking, particularly within dynamic and complex environments [1,2]. This involves identifying and following individual animals across scenes, including erratic movements, varying postures, and interactions with other animals or environmental elements. The complexity is further compounded by overlapping objects, occlusions, and visually cluttered backgrounds, which hinder the algorithm’s ability to consistently and accurately isolate and monitor the target animal or its particular feature [3].

The rapid advancements in machine learning (ML) and computer vision have underscored the critical importance of precise image segmentation as a precursor to robust model training and reliable predictive performance. Object-based segmentation, emphasizing the identification of distinct objects instead of individual pixels, has become a critical research focus. This approach is particularly valuable in complex and dynamic visual settings, where traditional pixel-based methods often fail to maintain object-specific details, resulting in reduced classification accuracy and higher computational demands. While advancements in machine learning have significantly enhanced image analysis, ensuring reliable and efficient segmentation in these contexts remains a persistent challenge. Previous studies have explored various preprocessing and segmentation techniques to address these challenges. Convolutional neural networks (CNNs) and transfer learning models have proven their efficacy in structured environments. However, the process often demands extensive computational resources, limiting their scalability for real-time applications [4,5,6]. On the other hand, unsupervised approaches, such as K-means clustering and principal component analysis (PCA), offer computational efficiency but lack the adaptability required for highly variable visual contexts [7,8,9,10]. While quantization and mathematical morphology have shown promise in simplifying image complexity and enhancing feature extraction, their integration into a cohesive preprocessing pipeline remains an open challenge [11,12].

In contrast to conventional methods that primarily depend on pixel-level modifications or computationally intensive processes, we propose an algorithm that combines convolutional operations, quantization strategies, and polynomial transformations to enhance segmentation accuracy while minimizing computational complexity. Such a move addresses a critical gap in literature by proposing an innovative preprocessing algorithm tailored for object-based image segmentation in complex visual environments. By generating metadata for each object, the framework enables enhanced interpretability and manageability of training datasets, providing a scalable solution for machine learning applications requiring precise object classification.

The current study aims to develop a systematic preprocessing pipeline that bridges the gap between computational efficiency and segmentation fidelity. The proposed approach aims to improve machine learning model performance in real-time and resource-constrained environments through adaptive object delineation and feature extraction. By addressing the limitations of existing methods, this research contributes a novel framework that paves the way for advancements in automated image processing and its application to high-stakes domains such as medical diagnostics, environmental monitoring, and autonomous systems.

## 2. Background

### 2.1. Image Preprocessing and Segmentation in Machine Learning

Image preprocessing plays a pivotal role in optimizing data quality, enhancing the accuracy of machine learning models, and ensuring robust image analysis workflows. Effective preprocessing techniques, such as image resizing, color adjustment, and noise reduction, enable the extraction of salient features, which are particularly critical in domains like medical imaging and autonomous systems [8,13,14]. These methods reduce computational overhead and standardize datasets, contributing to more reliable model performance.

Modern approaches frequently incorporate convolutional operations to normalize pixel intensities, thereby improving segmentation efficacy and the reliability of downstream predictions [15,16]. For instance, techniques such as histogram equalization have been widely applied to enhance contrast in low-light environments, while Gaussian smoothing is often used to suppress noise without compromising structural details [17].

Boundary delineation is another essential preprocessing step, with algorithms like the Canny edge detector and Sobel filter being commonly employed to extract structural contours critical for accurate segmentation [18,19]. This step ensures that models can identify object boundaries in diverse contexts, from biomedical imaging to remote sensing applications. Optimized preprocessing has been shown to significantly boost machine learning model performance in complex visual environments, such as those characterized by high object density, low contrast, or overlapping objects [6,20].

Advanced adaptive preprocessing techniques have further enhanced segmentation capabilities. As Zhou et al. [20] demonstrated, convolutional kernels tailored to the dataset’s specific characteristics can achieve high-fidelity segmentation in multidimensional data, enabling accurate feature extraction even in highly heterogeneous datasets. These approaches align with the broader shift toward computationally efficient preprocessing strategies that integrate seamlessly with sophisticated machine learning architectures, such as convolutional neural networks (CNNs) and transformers [21,22].

Furthermore, recent developments in unsupervised and semi-supervised learning have expanded the role of preprocessing in segmentation tasks. Techniques such as clustering-based preprocessing or generative adversarial networks (GANs) for data augmentation have proven effective in addressing data scarcity and improving segmentation performance across diverse applications, including agricultural monitoring and industrial inspection [23,24,25].

In summary, image preprocessing and segmentation form the foundation of effective machine learning pipelines for image analysis. By adopting adaptive and computationally efficient strategies, researchers can ensure robust model training and deployment across a wide range of challenging visual contexts.

### 2.2. Mathematical Morphology and Image Feature Extraction

**Figure 1:**
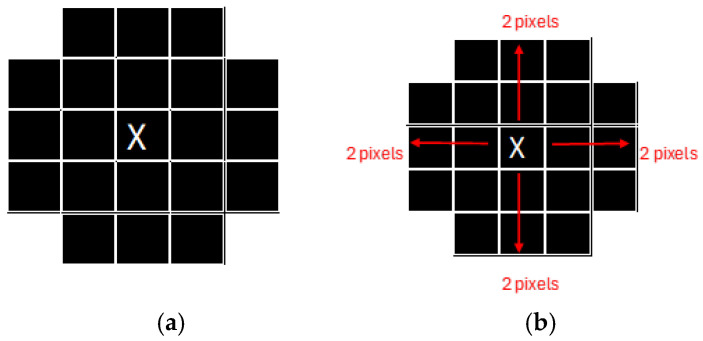
Example of the image pixels. (**a**) Example of the shape of the image (**b**). Source: Adapted from Ledda [31].

Mathematical morphology, utilizing operators such as dilation, erosion, and convolutional structuring elements, is fundamental in analyzing object shapes and sizes within images. As pioneered by Serra [26], morphological operations offer a systematic methodology for isolating salient image features, thereby refining segmentation and enhancing classification accuracy [27]. This technique has proven particularly valuable in delineating structures within medical images, underscoring its suitability for tasks demanding precise object identification [28]. Contemporary applications have successfully integrated morphological transformations with machine learning models, augmenting feature clarity and model interpretability [12]. This synergistic approach streamlines the workflow from feature extraction to model training, a process integral to the present study’s methodology [29]. Furthermore, novel methods combining morphological thresholding with contextual features demonstrate considerable promise in complex segmentation tasks, including ultrasound imaging [29] and optic disc detection [30]. Figure 1 represents the image and the shape definition calculation.

Mathematical morphology is a powerful framework for image segmentation and feature extraction, utilizing mathematical models to process and analyze the geometric and spatial structures within images. By defining shapes and leveraging set theoretical and topological operations, mathematical morphology enables precise identification and characterization of features in complex datasets.

For instance, as illustrated in Figure 1, the segmentation process begins with the definition of a target shape within the image. In Figure 1a, the target feature is identified by calculating pixel distances relative to a central pixel. Specifically, two pixels are measured from the bottom to the central pixel, two pixels from the central pixel to the top, and two pixels each from the east and west. This structured approach facilitates the accurate delineation of the desired feature’s shape.

**Figure 2:**
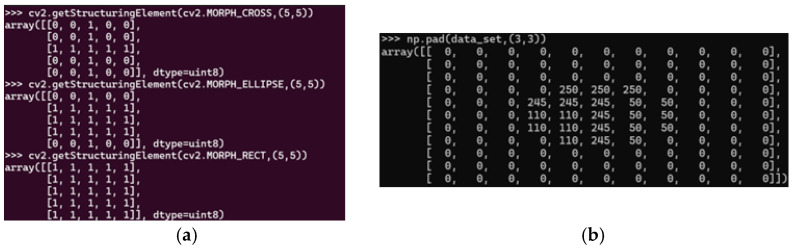
Cross, ellipse and rectangle morphology. (**a**,**b**) Original image. Sources: (**a**,**b**) the authors.

Another practical application of mathematical morphology involves leveraging morphological operations available in libraries such as OpenCV. As demonstrated in Figure 2, morphological operations can employ various structuring elements, including cross, elliptical, or rectangular shapes. These structuring elements determine the regions where morphological operations are applied, with pixels denoted by 1 indicating areas of operation and pixels denoted by 0 representing non-operational areas. The adaptability of these structuring elements enhances the flexibility and robustness of morphological analysis, allowing it to be tailored to a wide range of image-processing tasks.

These examples underscore the significance of mathematical morphology in improving the accuracy and efficiency of image segmentation, making it an indispensable tool in applications such as medical imaging, industrial inspection, and remote sensing. Figure 2 represents the structuring of cross, ellipse, and rectangle morphology and the image base to apply these morphologies.

In Figure 2, the structuring elements—cross, ellipse, and rectangle—are presented, with each element defining regions of interest for morphological operations. Pixels assigned a value of 1 denote areas where measurements or transformations will be applied, whereas pixels assigned a value of 0 indicate regions excluded from these operations. This binary representation forms the basis for precise and targeted feature analysis within images.

**Figure 3:**
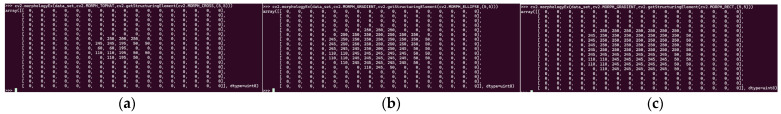
Image with cross morphology applied (**a**), image with ellipse morphology applied (**b**), and image with rectangle morphology applied (**c**). Sources: (**a**–**c**) the authors.

When these morphological structuring elements are applied to the original image, as demonstrated in Figure 3, the resulting transformations highlight specific spatial features and geometries. This approach allows for the effective segmentation and analysis of image components, adapting to the unique requirements of the dataset.

This example underscores the versatility and precision of mathematical morphology when used in conjunction with tailored structuring elements. By enabling targeted pixel-level analysis, this methodology enhances the robustness of image segmentation processes across diverse applications. Some structures of morphology are shown in Figure 3.

Figure 3 represents the application of cross, ellipse, and rectangle morphology.

As depicted in Figure 3, the application of different morphological structuring elements—cross, ellipse, and rectangle—results in distinct transformations of the original image. These transformations demonstrate the adaptability of mathematical morphology in tailoring image analysis to specific spatial patterns and features.

The benefits of employing morphology in image processing include its ability to enhance the extraction of geometric features and improve the segmentation of complex structures. For instance, cross morphology (Figure 3a) emphasizes linear and intersecting features, making it suitable for detecting narrow, grid-like patterns. Ellipse morphology (Figure 3b), on the other hand, is effective in isolating rounded or elliptical shapes, optimizing the segmentation of circular regions. Rectangular morphology (Figure 3c) is particularly advantageous for detecting elongated or rectangular structures, such as edges or contours.

These morphological operations provide a robust framework for preprocessing and feature extraction in machine learning workflows. By enabling targeted and adaptive image transformations, mathematical morphology facilitates improved accuracy in downstream classification and analysis tasks, especially in scenarios involving diverse and complex datasets.

### 2.3. Quantization Techniques in Image Processing

Quantization techniques reduce image complexity by clustering pixel values, allowing for efficient data processing while minimizing information loss. Among these techniques, K-means clustering is widely recognized for its effectiveness in simplifying image data [11]. Recent studies have shown that quantization reduces data volume and enhances feature visibility, which is essential in classification tasks [30]. This method enables the management of complex visual scenes by standardizing pixel representation, lowering computational requirements, and facilitating further model analysis [31]. Kalake et al. [32] combined quantization with feature labeling to optimize data preparation for segmentation, showcasing its potential in automated, large-scale image analysis. Additionally, advanced quantization methods, such as those incorporating cellular automata and quantum dots, have opened new avenues for nanoscale image processing [33], while innovative approaches in water-level recognition and object tracking have demonstrated the versatility of quantization across various applications [34,35].

### 2.4. Convolutional and Clustering Methods for Segmentation Enhancement

Convolutional and clustering methods play crucial roles in machine learning-based image segmentation, where convolutional operations help detect spatial hierarchies, and clustering techniques refine segmented areas. Convolutional networks filter image features effectively, establishing a foundation for robust image analysis [36]. Meanwhile, clustering techniques, such as K-means++, enhance differentiation within segmented regions, making these approaches valuable in medical and technical imaging [37]. Previous studies have confirmed that using convolutional and clustering methods together improves object localization and feature mapping accuracy, which is critical for applications requiring detailed segmentation, such as in robot-assisted surgery and regenerative medicine imaging [38,39]. The approach proposed in the current study employs a convolution-clustering approach, showcasing its potential to enhance segmentation accuracy, especially in complex image datasets characterized by high variability [39,40].

### 2.5. Applications of Image Processing in Machine Learning

Image processing is a cornerstone of automated object detection and classification in machine learning, enabling the extraction of meaningful patterns and features from visual data. Modern advancements, particularly in convolutional neural networks (CNNs) and transfer learning, have significantly improved the ability of models to identify intricate patterns within extensive datasets, enhancing performance across numerous domains [41,42].

In the medical field, image processing techniques have been instrumental in developing systems for early disease detection and diagnosis. Applications such as breast cancer prediction using mammographic images, leukemia classification through blood smear analysis, and brain imaging for Alzheimer’s detection highlight the critical role of image processing in improving healthcare outcomes [43,44]. Veterinary science also benefits from these advancements, with applications including livestock health monitoring [45,46].

Beyond healthcare, the applications of image processing extend into industrial, agricultural, and environmental domains. For instance, textile quality control utilizes image-based techniques for defect identification and fiber composition analysis [42,43]. In agriculture, image processing aids in tasks such as crop disease detection, weed identification, and yield prediction [46,47]. Meanwhile, remote sensing applications leverage high-resolution satellite imagery for urban planning, environmental monitoring, and disaster management [47].

The integration of preprocessing, quantization, and feature extraction methodologies in image processing pipelines has further expanded the practical scope of machine learning in complex visual data applications. These techniques optimize model training workflows, reduce computational overhead, and enhance interpretability, making them indispensable for handling high-dimensional and heterogeneous data [8].

Table 1 includes a list of applications by domain of uses of image processing and machine learning.

As illustrated in Table 1, image processing and machine learning demonstrate diverse applications across various domains. These include monitoring livestock health, behavior, and stress; advancing urban planning, infrastructure management, and traffic analysis through sophisticated image processing techniques; and contributing to medical fields by facilitating disease detection and the analysis of medical images for diagnosis and treatment planning.

## 3. Methods

**Figure 4:**
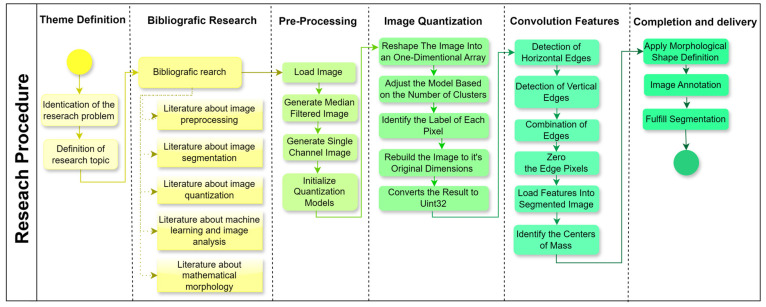
Flowchart of the overall research procedure. Source: the authors.

A structured research design was applied to the present study (Figure 4), progressing through six stages to address the research objectives effectively. Each stage was designed to ensure a systematic approach to image preprocessing, segmentation, and feature extraction, which is critical for enhancing machine learning applications in image analysis. The following sections outline each phase in detail, making the methodology replicable and aligned with the research question.

### 3.1. Theme Definition and Background

The initial stage involves identifying the research problem and defining the research topic. This step narrows the study’s scope by pinpointing specific challenges in automated image processing for machine learning applications. Through thoroughly examining existing research gaps, this phase establishes the research objectives and justifies the necessity of further investigation into advanced preprocessing and segmentation techniques. The definition of the research problem is guided by preliminary observations and trends in image processing applications, ensuring a clear foundation for subsequent stages. Our hypothesis is that it is possible to create a framework able to apply quantization and segmentation processes on images with complex backgrounds, such as images of cattle in nature.

**Figure 5:**
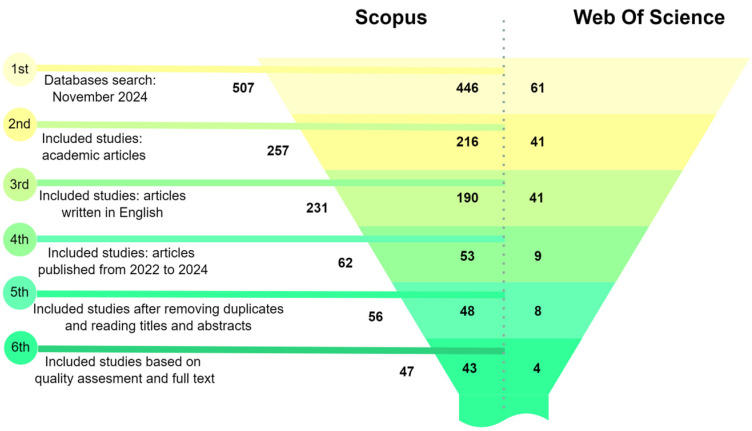
Literature review applied to the studied topics. Source: the authors.

Following the theme definition, a comprehensive literature review involves gathering the relevant literature on crucial topics, including image preprocessing, segmentation, quantization, machine learning in image analysis, and mathematical morphology. Sources are selected based on their contributions to these subfields, providing theoretical grounding and identifying established methodologies, tools, and unresolved challenges. The findings from the literature review informed the design of processing stages and helped justify the choice of analytical tools and approaches (Figure 5).

A systematic search of the Scopus and Web of Science databases was conducted using keywords related to image preprocessing and machine learning. An initial pool of 507 records, filtering by article type to include only peer-reviewed academic articles, was reduced to 257. Applying a language filter to select articles written in English further narrowed the selection to 231 articles. Restricting the publication date to the period between 2022 and 2024 yielded 62 articles, a timeline chosen to capture the most recent advancements in the field. After removing duplicates and conducting a detailed screening of titles and abstracts, 56 articles were subjected to a quality assessment. Ultimately, 47 articles were selected based on their relevance to the research topic. This systematic and rigorous selection process ensured a high-quality and focused dataset of studies, providing studies that met specific quality and relevance criteria in image preprocessing, segmentation, quantization, feature extraction, and mathematical morphology within the context of machine learning and image analysis.

### 3.2. Image Preprocessing

In the image preprocessing phase, contemporary methodologies often integrate convolutional operations to normalize pixel intensities, thereby enhancing segmentation accuracy and improving the reliability of downstream predictive tasks [15,16]. Furthermore, advanced adaptive preprocessing techniques have significantly improved segmentation performance, as highlighted by Zhou et al. [20].

In this context, a series of raw images are provided and undergo initial processing of pixel median calculation to generate an image with less noise and reduce the number of cores in the original image. A new image with a single-color channel was generated through an initial convolution from the image transmitted in memory to unify the channels to ensure better results, preparing the images for more advanced processing in subsequent stages. The initialization of all models was also performed at this stage. This step is crucial to standardizing input data and reducing core variability. Preprocessing procedures minimize potential biases in the data, such as lighting or image quality inconsistencies, which can influence the protection of segmentation and feature extraction.

### 3.3. Image Quantization

The image quantization process, as highlighted in the literature, plays a critical role in reducing data volume while enhancing the visibility of salient features, a crucial aspect of classification tasks [30]. K-means clustering, in particular, is widely acknowledged for its efficiency in simplifying image data by grouping similar pixels into clusters [11]. Additionally, Kalake et al. [32] demonstrate the integration of quantization with feature labeling as an effective strategy to optimize data preparation for segmentation, further improving the accuracy and efficiency of subsequent processing steps.

In this context, image quantization is a critical step in the preprocessing pipeline, aimed at reducing the complexity of visual data by limiting the number of unique colors while retaining the essential features required for subsequent machine learning tasks. The process begins with reshaping the image into a one-dimensional array, where the multidimensional pixel data are transformed into a single column with multiple rows. This adjustment standardizes the data structure, enabling efficient input into clustering models. The next step, fitting the model, involves defining the number of clusters that represent the desired palette of colors. The fitting process generates an array containing the selected colors or centroids, representing the image’s dominant tones.

Subsequently, each pixel is assigned a label corresponding to the nearest centroid through a prediction phase, effectively mapping the image to its reduced color space. The image is reconstructed to its original dimensions using these labels and centroids, ensuring that the spatial arrangement of pixels remains intact while adhering to the simplified color scheme. Finally, the processed image is converted into a positive integer format (Uint32), which optimizes computational storage and compatibility for downstream tasks.

### 3.4. Convolution Features

Convolutional neural networks effectively extract and filter image features, providing a robust foundation for advanced image analysis [36]. Their demonstrated potential to enhance segmentation accuracy is particularly significant in complex image datasets characterized by high variability [39,40].

In this context, convolution feature extraction underpins the algorithm, enabling region segmentation through edge detection and feature localization. Horizontal edges are emphasized using convolutional kernels to detect vertical pixel intensity changes, while vertical edges are highlighted by detecting horizontal variations. Merging these outputs provides a comprehensive representation of the image’s structural features.

Edge pixels are zeroed to isolate contiguous regions, creating well-defined segments by distinctly separating adjacent areas. Detected features are loaded into the segmented image, preserving spatial and contextual relationships, with centers of mass calculated and included as precise reference points for analysis or classification. This standardized approach ensures replicability through consistent convolutional operations and parameter settings while enhancing machine learning training via structured feature extraction. Potential biases from specific kernel designs are mitigated by diverse feature detection strategies, ensuring robustness across visual contexts. Integrating edge detection, segmentation, and feature localization provides a solid framework for interpreting visual data in complex environments.

### 3.5. Completion and Delivery

The final stage of the preprocessing algorithm consolidates the segmented features and prepares the processed data for machine learning applications by applying mathematical validations, annotations, and segmentation refinement. This phase ensures that each segment is accurately defined, annotated, and primed for iterative or subsequent processing if required.

The process begins with applying a morphological shape definition, which validates that each segment aligns with the desired mathematical representation of its shape. This validation uses the object’s center of mass as the origin point and accepts as parameters two kernels—one for the X-axis and one for the Y-axis. These kernels define the transformation required to confirm the segment’s adherence to its expected geometrical properties. Following this, the image annotation step is executed. A square with a specified radius is drawn around the feature’s center of mass, serving as an automated bounding box. This annotation provides a visual marker for the machine learning model, acting as a consistent reference point for feature learning and classification. The model gains a precise spatial context by leveraging these bounding boxes, enhancing its capacity to generalize and interpret the features in diverse visual scenarios.

The final step extracts regions of interest from the original image based on the validated segments defined in the preceding stage. This method returns a tuple containing the segmented regions, ensuring that the extracted data aligns with the algorithm’s predefined structure. If the segmentation results require refinement, the process can be restarted seamlessly, providing an iterative framework for optimization. This structured approach ensures that the completion and delivery stage align with the overarching research objectives and facilitates replicability and robustness in handling complex image environments. Biases, such as errors in kernel selection or segmentation boundaries, are mitigated through parameter flexibility and iterative validation, ensuring reliable delivery of high-quality data to downstream machine learning processes.

**Figure 6:**
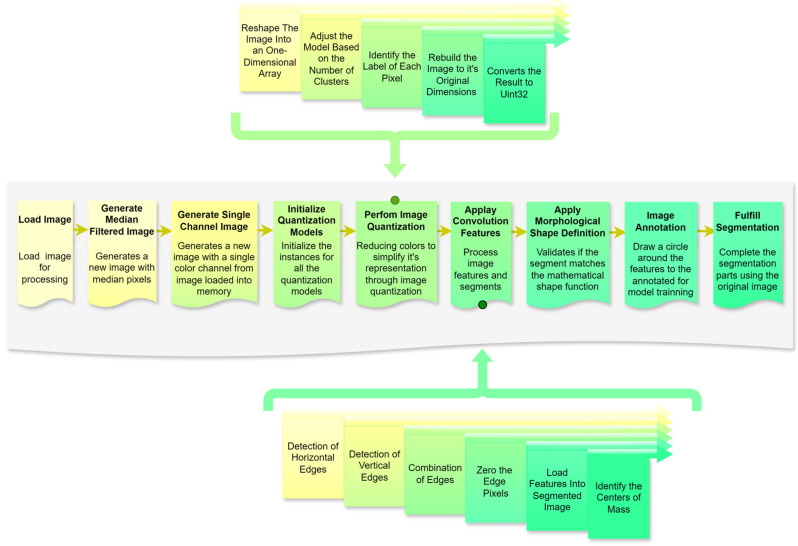
Schematic of the automated segmentation algorithm. Source: the authors.

Using libraries (SciPy, NumPy, OpenCV, Matplotlib, WebAgg, and Scikit-Learn), the stepwise methodology flow is shown in Figure 6. The automated segmentation algorithm’s pipeline integrates structured and automated processes to enable efficient image preprocessing, feature extraction, and segmentation. This methodology ensures high replicability and alignment with the research objectives, delivering a robust foundation for object-based image analysis in complex visual environments.

## 4. Results

The proposed preprocessing algorithm improved object segmentation, feature extraction, and computational efficiency across complex image backgrounds. The implementation employed multiple computational tools to facilitate image segmentation and feature extraction in complex backgrounds.

### 4.1. Load Image

**Figure 7:**
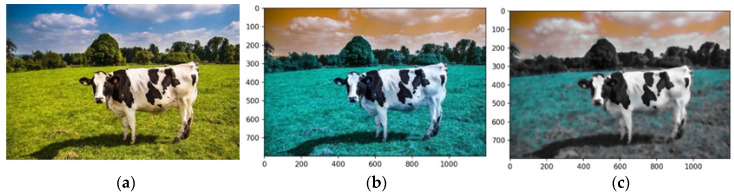
Original image from the UFMG website (**a**), image preprocessed using OpenCV’s imread function (**b**), and image preprocessed using BGR model (**c**). Sources: (**a**), Adapted from [48] UFMG—Veterinary College (2024); (**b**,**c**), created by the authors.

Each pipeline component uses an image of a cow (Figure 7a). The pipeline begins by loading the input image using robust image-handling libraries. This step ensures compatibility with downstream operations by standardizing input dimensions, resolution, and intensity distributions. Images are resized to a fixed resolution, and pixel intensity normalization is applied to create a consistent dataset for processing. These adjustments mitigate variability across input images, reducing preprocessing overhead.

Figure 7b shows the preprocessed image loaded via OpenCV’s function in the RGB color model. In contrast, Figure 7c uses the BGR model, highlighting the impact of color channel ordering on visual interpretation. Scale invariance was maintained by standardizing all images to ensure consistent feature extraction and analysis. These were reshaped into a tabular format using NumPy’s ‘reshape’ function, converting the 2D image array into a 1D array, where each row represents a pixel’s RGB values.

### 4.2. Generate Median-Filtered Image

A median filter was applied to the input image to reduce high-frequency noise while preserving edge details, enhancing object boundaries crucial for segmentation. Replacing each pixel’s intensity with the median value of its neighborhood eliminated slight isolated noise without compromising boundary integrity, as shown in Figure 7c.

A 3D median filter with a kernel size of (5,5,5) was used to process RGB images, ensuring noise reduction across all channels while maintaining inter-channel consistency. Unlike traditional 2D filters applied separately to each channel, this method minimizes artifacts and preserved color data integrity. The kernel size (5,5,5) balanced effective noise suppression with edge preservation, which is critical for segmentation, feature extraction, and classification. This configuration ensured robust processing by maintaining object details and reducing noise interference.

In this study, the kernel size of 5 × 5 × 5 was selected to align with the desired size of the features to be extracted. This choice directly relates to the dimensions of the features we aim to preserve and analyze. If smaller features are of interest, a smaller kernel size should be configured. Conversely, to focus only on features larger than 5 × 5 × 5 pixels, a larger kernel size should be set.

### 4.3. Generate Single-Channel Image

**Figure 8:**
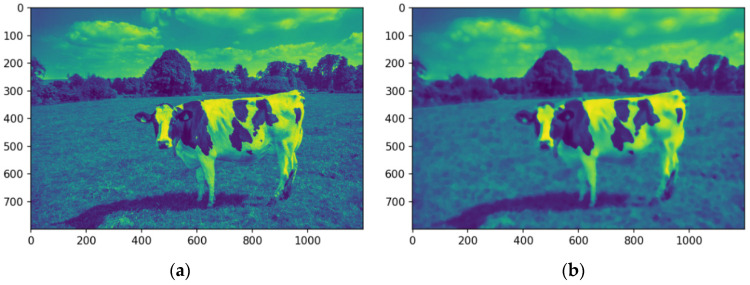
Images in one channel, original (**a**) and median-filtered (**b**). Source: the authors.

The image is converted into a single-channel representation to simplify computational requirements and streamline analysis. Selecting the most prominent or frequently occurring color channel shifts the focus to the features most pertinent to segmentation. This dimensionality reduction strategy enhances computational efficiency while preserving essential visual information. Figure 8a shows the image after the conversion into a single-channel representation, while Figure 8b illustrates the image using a median filter.

The analysis targets key visual features by isolating the dominant color channel. The image was linearized and reshaped into a two-dimensional array while maintaining the BGR channel structure, enabling the application of a computationally efficient single-channel convolution kernel. Dimensionality reduction preserves essential visual features while improving efficiency for segmentation. Figure 8 illustrates the original image (a), its single-channel representation, and the median-filtered version (b). The median-filtered image (b) proves more effective for segmentation, particularly in distinguishing the cow from the background by homogenizing grass texture, thereby enhancing object differentiation.

### 4.4. Initialize Quantization Models

A K-means clustering algorithm was employed to segment the image into discrete clusters based on color or intensity values. This process involves identifying representative centroids that define the clusters to which pixels are assigned. The model adapts to various image types and lighting conditions by initializing the K-means algorithm. We created two separate K-means instances: one for the original image and another for the median-filtered image. This study employed K-means clustering using Scikit-learn to reduce the image’s color palette and simplify subsequent processing.

In this module, the number of clusters was initialized to eight, corresponding to the total number of colors that the algorithm applies to the image following the quantization process. The algorithm is configured to partition the image pixels into eight distinct clusters by defining this hyperparameter as eight. Each cluster encapsulates a unique color or a specific range of similar colors, effectively reducing the image’s color palette while preserving its essential visual information.

### 4.5. Perform Image Quantization

**Figure 9:**
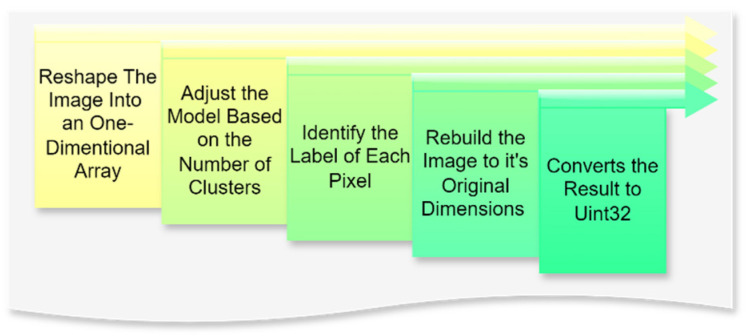
Flowchart of the performed image quantization. Source: the authors.

Image quantization is performed by mapping each pixel to the nearest cluster centroid using the initial model. This process reduces color or intensity variability across the image, simplifying the segmentation task while maintaining essential features. The resulting quantized image serves as a foundation for detecting meaningful object boundaries. Figure 9 presents the subprocess, including the performed image quantization.

A K-means clustering-based quantization technique was applied to reduce the image’s color palette and simplify subsequent analysis, involving the following steps in Table 2.

**Figure 10:**
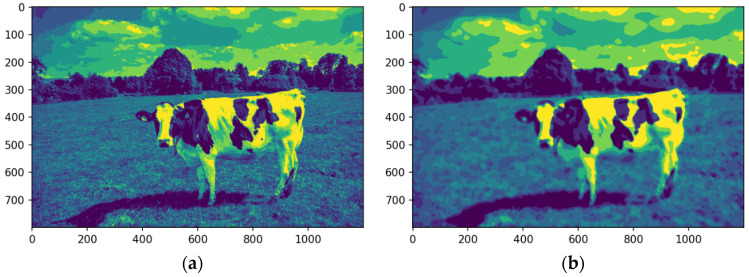
Image quantized, original (**a**) and median-filtered (**b**). Source: the authors.

Figure 10 illustrates the image after the quantization stage was performed.

After K-means quantization (Figure 10a,b), the original and median-filtered images were reduced to an eight-color palette. While both showed reduced color complexity, the median-filtered image exhibited a more explicit feature definition, notably enhancing the cow’s contours and its distinction from the background. This improvement was particularly evident in the sky and cloud regions, where quantization produced a more coherent representation. The process improved efficiency for subsequent tasks like segmentation and feature extraction by reducing the color palette while retaining key visual details.

### 4.6. Apply Convolution Features

**Figure 11:**
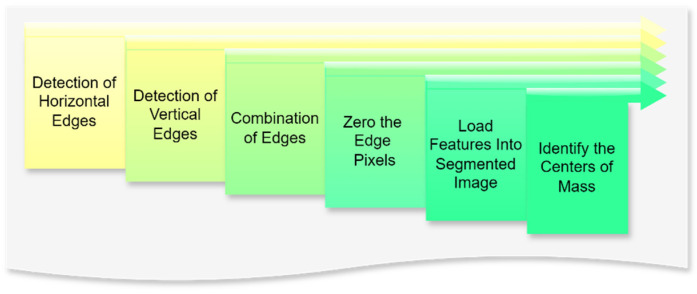
Flowchart of applying the convolution features. Source: the authors.

The pipeline employs convolutional operations to extract spatial features from the quantized image, such as edges and textures. Horizontal and vertical edge detection filters are applied to capture directional patterns, which are then merged to form a comprehensive feature map. These operations enhance the differentiation of objects within the image, enabling robust segmentation. Figure 11 presents the subprocess, including the applied convolution features.

**Figure 12:**
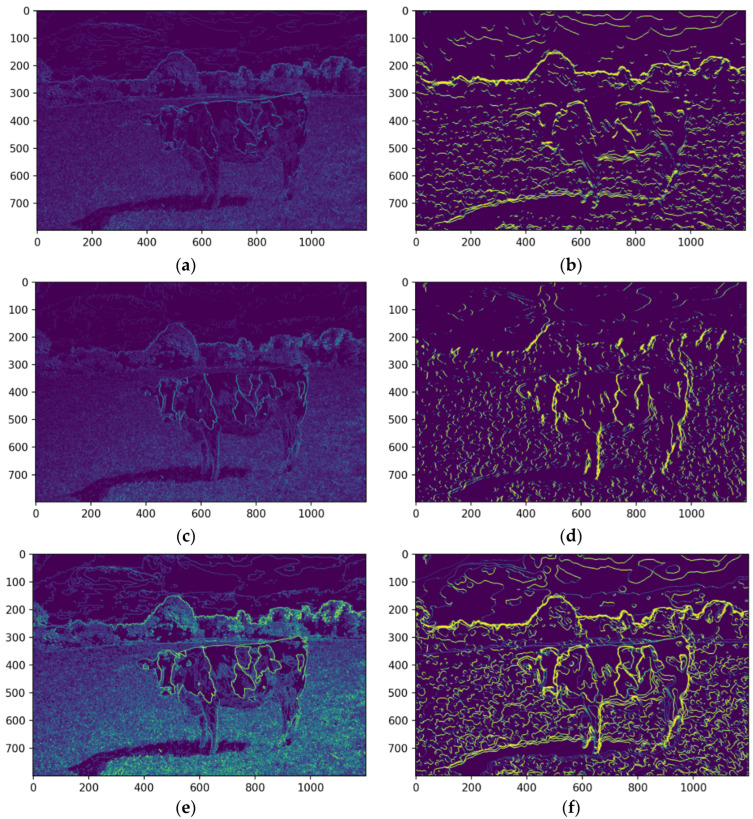
The horizontal feature detection of the original image (**a**) and median filtered (**b**), the vertical feature detection of the original image (**c**) and median filtered (**d**), and the combination of edges of the original (**e**) and the median filtered (**f**). Source: the authors.

A convolutional filter-based approach was employed to extract salient features from the image. As displayed, this technique involves applying filters to detect specific image patterns. A horizontal edge detection filter was applied to identify horizontal lines and edges within the image. This filter highlights areas of rapid intensity change in the horizontal direction (Figure 12a,b). The median-filtered image significantly enhances the detectability of horizontal edges, which are crucial for defining the contours of features. By reducing noise and smoothing the image, the median filter improves the signal-to-noise ratio, making it easier to identify and extract relevant features. This enhanced edge information facilitates more accurate segmentation and analysis of the image content.

A vertical edge detection filter was applied to detect vertical lines and edges. This filter emphasizes areas of rapid intensity change in the vertical direction. As illustrated in Figure 12c,d, the median-filtered image enhances the detectability of vertical edges, which are crucial for defining the contours of features. 

**Figure 13:**
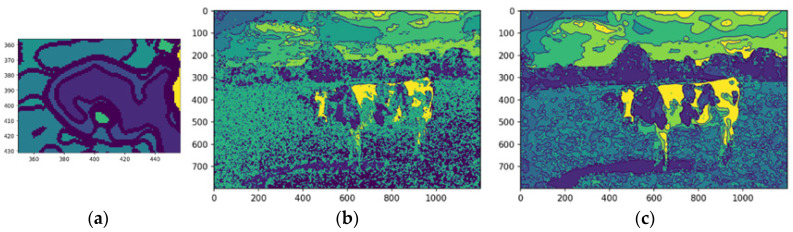
Representation of the edge pixel with zero (**a**) and the resulting center of mass identification in the original (**b**) and the median filtered (**c**). Source: the authors.

The outputs of the horizontal and vertical filters were merged into a single feature map, representing the image’s overall edge and line structure. This combined map (Figure 12e,f) comprehensively depicts structural features, enhancing edge and contour accuracy. Integrating these complementary edge maps enables the detection of complex shapes and patterns not evident in individual maps. Edge pixels were set to zero to isolate objects or regions of interest, creating clear segment boundaries. As shown in Figure 13a, this separation enhances segment distinction, minimizing interference from neighboring regions and improving the accuracy of tasks like segmentation and feature extraction.

Extracted features were integrated into the segmented image, creating a comprehensive representation for further analysis. Overlaying these features onto the original image highlights critical regions of interest, enabling tasks such as object recognition and scene understanding. The center of mass was calculated for each feature, providing a quantitative measure of spatial location for tasks like object tracking and shape analysis. The median-filtered image significantly enhanced the accuracy of these calculations (Figure 13b,c).

By enhancing feature delineation and reducing noise, the median filter enabled more precise localization of the center of mass, particularly for objects with complex shapes or indistinct boundaries. This improvement is evident in the more accurate representation of the cow’s center of mass in the median-filtered image, as the enhanced feature definition allows for a more reliable calculation.

**Figure 14:**
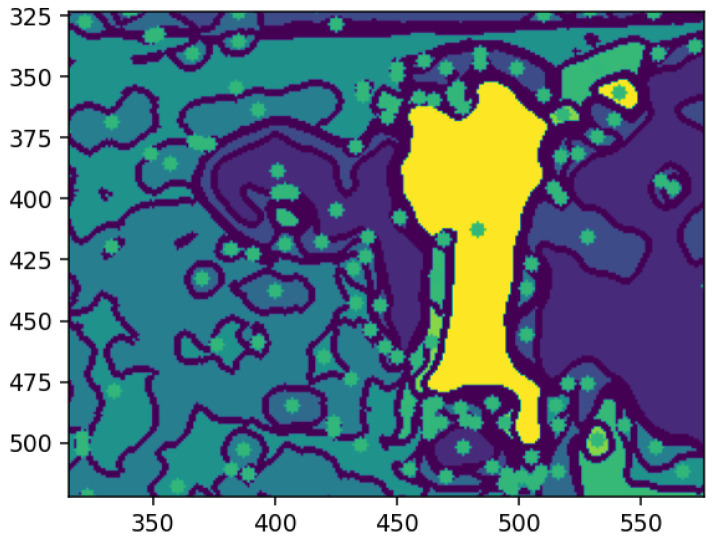
Center of mass on a specific feature. Source: the authors.

Figure 14 shows each feature annotated with a square marking its center of mass, the weighted average position of all pixels within the feature. This geometric representation clarifies spatial distribution, enabling the identification of relative positions and aiding tasks, like feature tracking, object recognition, and segmentation. Highlighting centers of mass provides a concise, computationally efficient representation for further analysis.

### 4.7. Applying the Morphological Shape Definition

**Figure 15:**
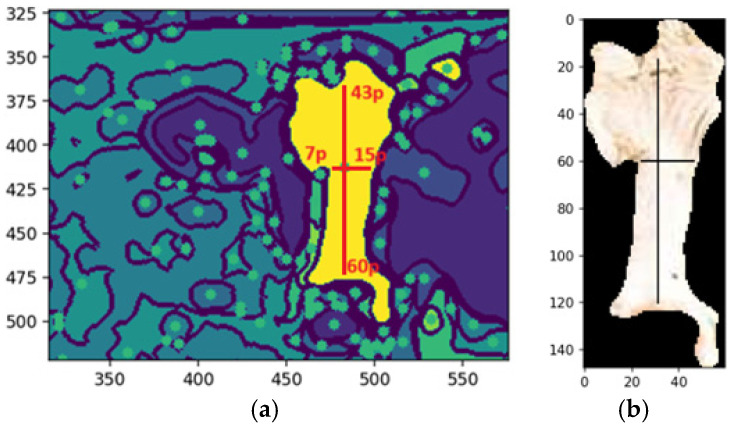
Shape of the forehead feature (**a**,**b**). Extraction of forehead based on the shape. Sources: (**a**,**b**) the authors.

A morphological validation step ensures that the detected segments represent valid objects by analyzing geometric properties, like shape, size, and center of mass, discarding non-conforming segments. The method uses the center of mass as the origin for mapping along the X and Y axes, guided by kernels defined as pixel ranges before and after the center. Morphological shape criteria determine whether a segment matches the expected characteristics, returning a Boolean value: valid if it aligns with the target feature’s morphology, otherwise invalid. This process filters noise and irrelevant regions, ensuring segmentation integrity. Figure 15a shows an example of the dimensions of the shape of the front head feature, and Figure 15b shows the extraction of the feature with the same dimensions of shape.

Figure 15a illustrates an example of the morphological shape definition applied to identify a specific feature on the forehead of a cattle. In this case, the feature’s center of mass, determined by the central pixel, served as the reference point for defining its spatial dimensions. The region of interest was delineated by extending 43 pixels upward and 60 pixels downward along the y-axis, with 7 pixels to the left and 15 pixels to the right along the x-axis. This precise bounding allowed for accurate identification and isolation of the feature, demonstrating the effectiveness of the morphological shape definition technique in segmentation tasks, while Figure 15b illustrates the segmented feature based on the shape definition.

The desired pattern is defined based on the feature’s center of mass to detect a specific feature, such as a cow’s forehead. From this central point, pixel extensions to the boundary are calculated in all cardinal directions—up, down, left, and right—to delineate the feature’s structure. The method scans the image for regions matching the defined morphology. If a segment, such as the belly instead of the forehead, does not align with the target pattern, it is deemed invalid. This validation step ensures that only features matching the defined shape advance to further processing (Figure 15).

Morphological shape definition in segmentation enhances precision, efficiency, and feature extraction accuracy. Specifying the expected shape of the cow’s forehead ensures that only relevant segments are selected, reducing false positives by filtering out irrelevant regions like the neck or body. This approach provides an apparent geometric reference, improving robustness and enabling reliable distinction of the forehead from similar features. It also optimizes computational efficiency by focusing analysis on pertinent regions, reducing processing time and computational costs while supporting tasks like feature tracking and classification.

Validated segments were annotated with bounding boxes, using the center of mass as the reference point. These annotations delineate the feature’s boundaries, aiding machine learning models in interpreting the location and shape for object recognition and classification. They also provide visual feedback for validating segmentation accuracy and enhance model training by supplying labeled data, improving generalization to unseen images and optimizing pipeline performance.

**Figure 16:**
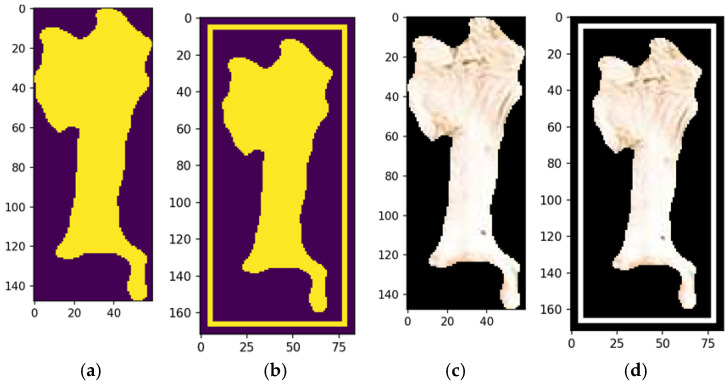
Morphological shape definition of a feature (**a**), automated bounding box (**b**), fulfill segmentation and bounding box options—no (**c**) and yes (**d**). Source: the authors.

Figure 16b shows the automated bounding box precisely delineating the cow’s forehead, streamlining object detection by consistently identifying and annotating the region of interest. This provides a clear spatial representation for tasks like classification or tracking. Automated bounding boxes reduce manual annotation, saving time and effort while enhancing pipeline efficiency. They ensure uniformity and reproducibility across datasets, supporting scalable and reliable machine learning training. In Figure 15b, the bounding box serves as both a visual aid and a precise reference, improving the accuracy and effectiveness of subsequent processing steps.

The final stage refines the segmentation output, ensuring coherence and completeness through morphological operations that resolve overlaps and gaps, creating a comprehensive segmentation map for downstream tasks. This method returns a tuple of valid image regions defined by coherent, non-overlapping segments from the refinement process (Figure 16c). Each tuple includes spatial boundaries and associated features, preserving segmentation integrity and compatibility with further steps. The process allows for iterative adjustments, enabling parameter refinement or addressing issues like over- or under-segmentation, improving accuracy and robustness for complex or application-specific data (Figure 16d).

The fulfill segmentation method enhances segmentation adaptability and precision by returning a tuple of high-quality, coherent image regions, reducing noise and errors in subsequent analyses. Optional bounding boxes provide flexibility for spatial localization, or a generalized segmentation map based on task requirements. The method’s iterative capability allows for parameter adjustments and tailored refinements, addressing challenges like irregular boundaries or complex images. This flexibility improves segmentation accuracy, robustness, and scalability, making it suitable for diverse applications, such as object recognition, pattern analysis, and machine learning data preparation.

**Figure 17:**
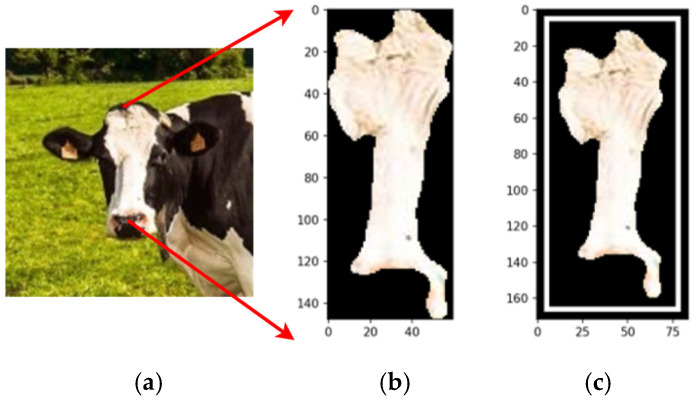
The original image (**a**), the segmentation (**b**), and the feature segmented (**c**). Source: the authors.

Figure 17 compares the original cow photograph (a) with the segmented feature (b), where the detected feature is highlighted in its original color, preserving detail. This segmentation isolates relevant features precisely, enabling accurate analysis while retaining critical information for further classification.

**Figure 18:**
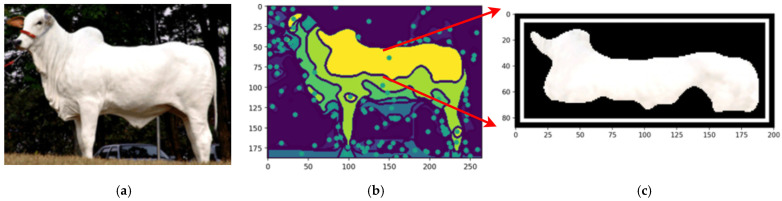
Original image of Nelore Cattle (**a**), centers of mass of the image (**b**), and the feature segmented (**c**). Sources: (**a**) Adapted from [49] Nelore Breeders Association of Brazil (2024) and (**b**,**c**), created by the authors.

By employing the proposed automated segmentation algorithm, as illustrated in Figure 3, the segmentation process yielded significantly enhanced results, even for cattle breeds with uniform coat colors. Texture-less breeds, such as Nelore (Figure 17) and Black Angus (Figure 18), present inherent challenges due to the lack of distinct patterns or markings. Despite these difficulties, the framework demonstrated its robustness by effectively identifying features through variations in color pigmentation. Even in texture-less breeds, subtle pigmentation differences enabled accurate detection of features, as evidenced by the calculated centers of mass. This performance underscores the framework’s adaptability to a wide range of visual complexities.

Figure 18 shows an example using a texture-less breed Nelore to capture the segmentations.

**Figure 19:**
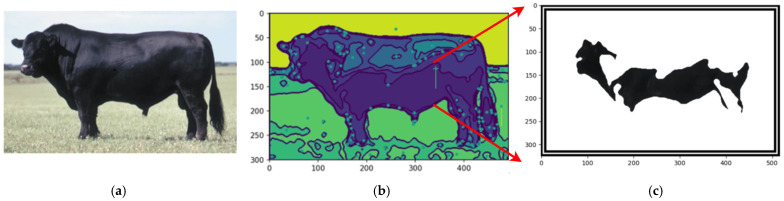
Original image of Black Angus (**a**), centers of mass of the image (**b**), and the feature segmented (**c**). Sources: (**a**) Adapted from [50] Angus (2024) and (**b**,**c**), created by the authors.

Figure 19 shows an example using a texture-less breed Black Angus to capture the segmentations.

The proposed automated segmentation algorithm offers several notable benefits, particularly in the context of livestock monitoring and management. Its ability to effectively handle texture-less breeds, such as Nelore and Black Angus, addresses a critical challenge in image analysis, where uniform coat colors typically hinder feature extraction. By leveraging subtle differences in pigmentation, the framework ensures precise and automated segmentation, enabling accurate identification of key features like the centers of mass.

Additionally, the algorithm’s robustness across diverse visual conditions highlights its potential for scalability and integration into broader applications, such as drone-based livestock monitoring and precision agriculture initiatives.

## 5. Discussion

The findings of this study underscore the significant advancements achieved by the proposed preprocessing algorithm in addressing the challenges of object-based segmentation in complex visual environments. The algorithm effectively delineates objects while maintaining computational efficiency by integrating convolution operations, quantization techniques, and polynomial transformations. The results demonstrate that this approach enhances segmentation accuracy and reduces computational overhead, paving the way for broader adoption of machine learning (ML) models in resource-constrained and real-time applications. Such an approach represents a significant step in bridging the gap between computational feasibility and the demand for high-fidelity object segmentation, a persistent challenge in computer vision.

The proposed algorithm demonstrates superior performance in several key areas compared with the existing literature. Traditional methods, such as CNN-based segmentation or transfer learning, often rely heavily on pixel-level adjustments, which, while effective in controlled scenarios, become computationally prohibitive in real-time or large-scale applications [6]. Similarly, while computationally efficient, unsupervised methods like K-means clustering fail to adapt to dynamic backgrounds and object variability [8]. The innovation of the current study lies in its ability to synergize these approaches, employing quantization to simplify image complexity and convolutional operations to enhance feature extraction, thereby offering a balanced solution that is both scalable and accurate. This improvement aligns with recent findings, emphasizing the need for preprocessing pipelines capable of handling diverse and intricate visual datasets [11].

Another contribution of this research is its capacity to generate structured metadata alongside segmented images. Unlike traditional methods focusing solely on segmentation, the proposed framework produces metadata that enhances the interpretability and reusability of the segmented objects. This advancement is particularly beneficial for applications requiring dynamic object classification, such as autonomous systems and medical diagnostics, where interpretability and accuracy are paramount. The algorithm improves classification performance by treating each object as an independent training unit. It supports iterative model refinement, aligning with calls in the literature for more modular and adaptive preprocessing techniques [2].

Despite its advancements, the algorithm has certain limitations. It relies on the initial calibration of convolutional and quantization parameters, which vary based on dataset complexity and application and require manual intervention. The future integration of adaptive parameter optimization, such as reinforcement learning or dynamic kernel adjustments, could address this challenge. Furthermore, while this study focused on static image datasets, extending the framework to video or temporal datasets remains an area for further research. Another limitation of these algorithms lies in their initiation of processing static images.

Overcoming this constraint could significantly enhance the algorithm’s versatility and scalability, paving the way for its application in dynamic contexts such as video analytics and real-time monitoring systems. A potential solution to address this limitation involves upgrading the process to automate the segmentation of video frames, thereby expanding the framework’s applicability to more complex and real-time scenarios.

Overall, the findings highlight the practical and theoretical contributions of the proposed preprocessing pipeline. By combining computational efficiency with high segmentation fidelity, this research provides a scalable and effective solution for object-based segmentation in machine learning. The implications of this work extend to various fields, including environmental monitoring, autonomous vehicles, and quality control in manufacturing, offering a robust foundation for future innovations in image preprocessing and analysis. Its practical implications are vast, spanning domains, such as autonomous systems, medical diagnostics, environmental monitoring, and manufacturing quality control.

As shown in Figure 13, the developed approach is essential in complex image processing tasks, where the ability to retain fine details can significantly enhance the performance of machine learning models, leading to improved accuracy and robustness in subsequent analyses. Such high-fidelity feature detection is crucial to advancing automated systems for object recognition and analysis, particularly in Precision Livestock Farming.

To implement the framework illustrated in Figure 6 using the Python programming language, the process involves a sequence of structured steps, each employing specific libraries and methodologies to achieve robust image processing and segmentation.

The first step involves loading the input image for processing. This can be achieved using widely adopted Python libraries such as OpenCV or PIL, which provide versatile functions for reading and handling image data. Once the image is loaded, the next step applies a median filter to reduce noise while preserving edge details. This operation is crucial for enhancing object boundaries and can be efficiently executed using the OpenCV library through its medianBlur function.

Subsequently, the multi-channel RGB image is converted into a single-channel image to simplify subsequent processing steps. This can be performed using the OpenCV cvtColor function, which transforms the image representation while retaining essential structural information.

To prepare the image for quantization, clustering algorithms such as k-means are utilized to categorize pixel values into distinct clusters. This step requires specifying the number of clusters, which determines the quantization level. For instance, in this study, the number of clusters was set to eight, effectively reducing the image to a palette of eight colors. The quantization process itself involves replacing each pixel’s value with the corresponding cluster centroid, thereby simplifying the image while maintaining critical features. This can be implemented using the KMeans module from the scikit-learn library.

Feature extraction is a pivotal step for identifying patterns and objects within the image. This can be achieved using convolutional operations, such as edge detection filters, available in OpenCV through functions like Sobel or Canny. These filters highlight the transitions in intensity, delineating object boundaries and facilitating segmentation.

Mathematical morphology is then applied to validate segments and refine identified features or patterns. This involves the use of morphological operations, such as closing or opening, to enhance or suppress specific structures within the image. The OpenCV morphologyEx function is particularly suited for this purpose, allowing for the precise manipulation of shapes based on structural elements.

Annotations are added around identified features or regions of interest to facilitate visualization and further analysis. Circles or bounding boxes can be drawn using functions such as circles or rectangles from the OpenCV library. Finally, segmentation is completed by mapping the processed features back onto the original image. This is achieved through bitwise operations, such as OpenCV’s bitwise_and, which merge segmented masks with the input image to produce the final segmented output.

By leveraging Python libraries such as OpenCV and scikit-learn, the implementation of this framework ensures a systematic and reproducible workflow. Each step is designed to enhance the quality and interpretability of the processed image, providing a robust foundation for feature extraction, segmentation, and subsequent analyses.

To conclude this manuscript, the implementation of the proposed framework integrates advanced Python libraries and tools to streamline image preprocessing, segmentation, and feature extraction for machine learning applications. The implementation leverages key libraries and their respective modules to create a modular, scalable, and efficient system. For instance, numpy handles array management, while scipy provides robust statistical, convolutional, and optimization functions. Machine learning functionalities are integrated using sklearn, and data visualization is facilitated through matplotlib.pyplot.

Image processing is managed with OpenCV (cv2), while dynamic module handling and asynchronous execution are supported by importlib and asyncio, respectively. The clustering and quantization processes are built on KMeans from sklearn, ensuring robust and adaptable data segmentation capabilities. By combining these tools, the framework achieves efficient preprocessing, accurate segmentation, and reliable feature extraction, laying a strong foundation for applications in complex datasets, such as animal behavior analysis and precision livestock farming. This implementation demonstrates the versatility of the framework and its potential to address a wide range of challenges in image-based machine learning workflows.

## 6. Conclusions

This study introduces a novel preprocessing algorithm tailored for object-based image segmentation in complex visual environments, addressing a critical gap in machine learning (ML) applications. The algorithm integrates convolution operations, quantization techniques, and polynomial transformations to achieve precise object delineation while maintaining computational efficiency. The proposed method enhances interpretability, facilitates data management, and improves ML training performance by systematically generating structured metadata alongside segmented images. These contributions significantly advance computer vision, particularly for applications demanding high segmentation fidelity under challenging visual conditions.

## Figures and Tables

**Table 1:** Applications by domain of uses of image processing and machine learning.

Description	References
Articles exploring machine learning and image processing applications in monitoring livestock health, behavior, and stress.	[1,3,7,12,15]
Research focused on classifying, segmenting, and analyzing agricultural products like fruits, vegetables, and herbs using ML techniques.	[4,8,11,16,19]
Papers that discuss the role of image processing in detecting diseases or analyzing medical images for diagnosis and treatment planning.	[2,5,9,13,17]
Studies on the use of machine learning to monitor wildlife, assess ecological conditions, or detect environmental changes.	[6,10,14,18,20]
Studies on the use of machine learning to monitor wildlife, assess ecological conditions, or detect environmental changes.	[21,24,26,29,30]
Articles with focus on urban planning, infrastructure management, or traffic analysis using advanced image processing techniques.	[22,23,27,28,31]

**Table 2:** The steps that were applied to reduce the image’s color palette and simplify subsequent analysis.

Step	Description
Reshape the image into a one-dimensional array.	The image is reshaped into a one-dimensional array, forming a single column of pixel values. This linearization optimizes processing for the K-means algorithm, enabling efficient clustering of pixel values into distinct groups while ensuring accuracy and scalability in the image processing pipeline.
Adjust the model based on the number of clusters.	Initially, with a predefined number of clusters, the K-means algorithm was trained on the reshaped image data to identify centroids representing color clusters. This process was applied to both original and median-filtered images, enabling a comparative analysis to assess the impact of median filtering on clustering performance.
Identify the label of each pixel.	Each pixel is labeled based on the nearest centroid identified during clustering, categorizing it into a predefined color group. This process quantizes the image’s color space, reducing it to representative colors, simplifying interpretation, and enabling efficient comparison of results across preprocessing methods, such as median filtering versus the original image.
Rebuild the image to its original dimensions.	The image is reconstructed by mapping each pixel’s label to its centroid color, restoring spatial structure with a reduced color palette. This quantization simplifies the image while preserving critical visual features, enabling efficient analysis of color patterns and comparison between preprocessing techniques, such as the original and median-filtered images.
Convert the result to uint32.	The quantized image is converted to a 32-bit unsigned integer (Uint32) data type to ensure compatibility with subsequent processing steps. This conversion allows for a precise representation of the reduced color palette.

## Data Availability

Data will be available upon request to the corresponding author.

## References

[1] Fang C., Zhang T., Zheng H., Huang J., Cuan K. (2020). Pose Estimation and Behavior Classification of Broiler Chickens Based on Deep Neural Networks. Comput. Electron. Agric..

[2] Chen Y., Jiao T., Song J., He G., Jin Z. (2024). AI-Enabled Animal Behavior Analysis with High Usability: A Case Study on Open-Field Experiments. Appl. Sci..

[3] Da Silva Santos A., De Medeiros V.W.C., Gonçalves G.E. (2023). Monitoring and Classification of Cattle Behavior: A Survey. Smart Agric. Technol..

[4] Cambra A.B., Muñoz A., Murillo A.C., Ollero A., Sanfeliu A., Montano L., Lau N., Cardeira C. (2018). How to Transfer a Semantic Segmentation Model from Autonomous Driving to Other Domains?. Proceedings of the ROBOT 2017: Third Iberian Robotics Conference. ROBOT 2017.

[5] Gherardini M., Mazomenos E., Menciassi A., Stoyanov D. (2020). Catheter segmentation in X-ray fluoroscopy using synthetic data and transfer learning with light U-nets. Comput. Methods Programs Biomed..

[6] Jemimma T.A., Vetharaj Y.J. (2022). Fractional probabilistic fuzzy clustering and optimization based brain tumor segmentation and classification. Multimed. Tools Appl..

[7] Liu L., Wang L. A Scalable Unsupervised Feature Merging Approach to Efficient Dimensionality Reduction of High-Dimensional Visual Data. Proceedings of the 2013 IEEE International Conference on Computer Vision.

[8] Morales-Vargas E., Padilla-Martinez J.P., Peregrina-Barreto H., Garcia-Suastegui W.A., Ramirez-San-Juan J.C. (2022). Adaptive feature extraction for blood vessel segmentation and contrast recalculation in laser speckle contrast imaging. Micromachines.

[9] Rahoma A., Imtiaz S., Ahmed S. (2023). Detection and diagnosis of process fault using unsupervised learning methods and unlabeled data. Int. J. Adv. Eng. Sci. Appl. Math..

[10] Yang M., Li M., Huang C., Zhang R., Liu R. (2024). Exploring the InSAR Deformation Series Using Unsupervised Learning in a Built Environment. Remote Sens..

[11] Xu C., Jia W., Wang R. (2023). MorphText: Deep morphology regularized accurate arbitrary-shape scene text detection. IEEE Trans. Multimed..

[12] Anitha J., Kalaiarasu M. (2022). MRI brain tumor segmentation with intuitionist possibilistic fuzzy clustering and morphological operations. Comput. Syst. Sci. Eng..

[13] Bhutto J., Tian L., Du Q., Sun Z., Lubin Y., Tahir M. (2022). CT and MRI Medical Image Fusion Using Noise-Removal and Contrast Enhancement Scheme with Convolutional Neural Network. Entropy.

[14] Saponara S., Elhanashi A. (2022). Impact of Image Resizing on Deep Learning Detectors for Training Time and Model Performance. Applications in Electronics Pervading Industry, Environment and Society.

[15] Wisaeng K. (2022). Breast cancer detection in mammogram images using K-means++ clustering based on cuckoo search optimization. Diagnostics.

[16] Pokkuluri K.S., Devi S.S.S.N.U., Margala M., Chakrabarti P. (2025). Enhancing Image Segmentation Accuracy using Deep Learning Techniques. J. Adv. Res. Appl. Sci. Eng. Technol..

[17] Yueyang G., Jinhui Z., Siyi W., Zheng L. (2024). PFormer: An efficient CNN-Transformer hybrid network with content-driven P-attention for 3D medical image segmentation. Biomed. Signal Process. Control..

[18] Kanikar M., Supak P. (2024). Hybrid CG-Like Algorithm for Nonlinear Equations and Image Restoration. Carpathian J. Math..

[19] Yu Y., Wang C., Fu Q., Kou R., Huang F., Yang B., Yang T., Gao M. (2023). Techniques and Challenges of Image Segmentation: A Review. Electronics.

[20] Zhou F., Chen B., Chen X., Han H. (2022). Neuronal morphological model-driven image registration for serial electron microscopy sections. Front. Hum. Neurosci..

[21] Mohd IM A., Firdaus A.M., Hafizi RM N.K., Baharuddin I., Shuhada R.A., Kamarol J.M., Syazwani M.N., Abdullahi M.A., Firdaus M.S. (2024). Performance Evaluation of Edge-Based Segmentation Methods for Electrical Tree Image Analysis in High-Voltage Experiments. J. Adv. Res. Appl. Sci. Eng. Technol..

[22] Amir M., Mina J. (2025). Unsupervised feature selection using sparse manifold learning: Auto-encoder approach. Inf. Process. Manag..

[23] Elakkiya R., Harshiv C., Nick P., Subramaniyaswamy V., Ketan K. (2024). Lung image quality assessment and diagnosis using generative autoencoders in unsupervised ensemble learning. Biomed. Signal Process. Control.

[24] Mandle A.K., Sahu S.P., Gupta G. (2022). Brain tumor segmentation and classification in MRI using clustering and kernel-based SVM. Biomed. Pharmacol. J..

[25] Shyamala B., Brahmananda S.H. (2023). Brain tumor classification using optimized and relief-based feature reduction and regression neural network. Biomed. Signal Process..

[26] Serra J. (1982). Image Analysis and Mathematical Morphology.

[27] Madathil S., Padannayil S.K. (2022). MC-DMD: A data-driven method for blood vessel enhancement in retinal images using morphological closing and dynamic mode decomposition. J. King Saud Univ.-Comput. Inf. Sci..

[28] Chugh S., Goyal S., Pandey A., Joshi S. (2022). Morphological and Otsu’s technique based mammography mass detection and deep neural network classifier based prediction. Trait. Du Signal.

[29] Tong G., Jiang H., Shi T., Han X.-H., Yao Y.-D. (2023). A lightweight network for contextual and morphological awareness for hepatic vein segmentation. IEEE J. Biomed. Health Inform..

[30] Fallahdizcheh A., Laroia S., Wang C. (2023). Sequential active contour based on morphological-driven thresholding for ultrasound image segmentation of ascites. IEEE J. Biomed. Health Inform..

[31] Ledda A. (2007). Mathematical Morphology in Image Processing. Ph.D. Thesis.

[32] Silva A.B., Martins A.S., Tosta T.A.A. (2022). Computational analysis of histological images from hematoxylin and eosin-stained oral epithelial dysplasia tissue sections. Expert Syst. Appl..

[33] Jin Y., Yu C., Yin J., Yang S.X. (2022). Detection method for table grape ears and stems based on a far-close-range combined vision system and hand-eye-coordinated picking test. Comput. Electron. Agric..

[34] Kalake L., Dong Y., Wan W., Hou L. (2022). Enhancing detection quality rate with a combined HOG and CNN for real-time multiple object tracking across non-overlapping multiple cameras. Sensors.

[35] Dou G., Chen R., Han C., Liu Z., Liu J. (2022). Research on water-level recognition method based on image processing and convolutional neural networks. Water.

[36] Prasenan P., Suriyakala C.D. (2022). Fish species classification using a collaborative technique of firefly algorithm and neural network. EURASIP J. Adv. Signal Process..

[37] Wisaeng K. (2022). CFLHCF: Simultaneous detection of the optic disc and exudates using color features, local homogeneity and contextual features. Trait. Du Signal.

[38] Guan B., Zou Y., Zhao J., Pan L., Yi B., Li J. (2023). Clean visual field reconstruction in robot-assisted laparoscopic surgery based on dynamic prediction. Comput. Biol. Med..

[39] Wang D., Yang S., Guo K.-X. (2023). Computer-aided recognition and assessment of a porous bioelastomer in ultrasound images for regenerative medicine applications. Med. Nov. Technol. Devices.

[40] Wang T., Dai Q. (2023). SURVS: A Swin-Unet and game theory-based unsupervised segmentation method for retinal vessel. Comput. Biol. Med..

[41] Zong W., Li M., Li G., Wang L., Wang L., Zhang F. (2023). Toward efficient and complete line segment extraction for large-scale point clouds via plane segmentation and projection. IEEE Sens. J..

[42] Monika, Bansal D., Passi A. (2022). Image forgery detection and localization using block based and key-point based feature matching forensic investigation. Wirel. Pers. Commun..

[43] Kirola M., Memoria M., Dumka A. (2023). Optimized U-Net convolutional neural network based breast cancer prediction for accuracy increment in big data. Concurr. Comput. Pract. Exp..

[44] Ramya V.J., Lakshmi S. (2023). An efficient hybrid model for acute myeloid leukemia detection using convolutional Bi-LSTM based recurrent neural network. Comput. Methods Biomech. Biomed. Eng. Imaging Vis..

[45] Abdelkader M. (2022). MATLAB algorithms for diameter measurements of textile yarns and fibers through image processing techniques. Materials.

[46] Xu X., Fan C., Wang L. (2022). A deep analysis of the image and video processing techniques using nanoscale quantum-dots cellular automata. Optik.

[47] Khetavath S., Sendhilkumar N.C., Mukunthan P., Jana S., Gopalakrishnan S., Malliga L., Chand S.R., Farhaoui Y. (2023). An intelligent heuristic manta-ray foraging optimization and adaptive extreme learning machine for hand gesture image recognition. Big Data Min. Anal..

[48] UFMG Veterinary College Saiba o Que é Vaca Louca e se a Doença Animal Pode Afetar o Ser Humano. https://vet.ufmg.br/clipping/saiba-o-que-e-vaca-louca-e-se-a-doenca-animal-pode-afetar-o-ser-humano/.

[49] Nelore Breeders Association of Brazil. www.nelore.org.br.

[50] Black Angus Bull. https://www.britannica.com/animal/Angus-breed-of-cattle.

